# AI-assisted radiomics for classification of benign and non-benign right heart masses in 2D-echocardiography

**DOI:** 10.1186/s12967-026-08322-8

**Published:** 2026-05-23

**Authors:** Yan Chen, Mengqing Deng, Linyuan Xie, Huiling Cheng, Jiali Wu, Anqi Yang, Yun Mou, Shenjiang Hu

**Affiliations:** 1 Echocardiography and Vascular Ultrasound Center, The First Affiliated Hospital, Zhejiang University School of Medicine, Hangzhou, China; 2 Department of Cardiology, The First Affiliated Hospital, Zhejiang University School of Medicine, No.79, Qing-Chun Road, Hangzhou, 310003 China

**Keywords:** Right heart masses, Echocardiography, Differential diagnosis, Machine learning, Deep learning

## Abstract

**Background:**

The rarity of right heart masses challenges diagnostic proficiency, while reproducibility is affected by the echocardiography operator. Artificial intelligence (AI)-based imaging tools may help address these limitations.

**Methods:**

In this retrospective study (2013–2024), we enrolled surgical patients with right heart masses and obtained preoperative transthoracic (TTE) and transesophageal (TEE) echocardiographic images. Two-dimensional (2D) TTE (*n* = 98) and TEE (*n* = 87) images underwent radiomics analysis. Binary classification models were developed to differentiate benign from non-benign lesions using five machine-learning (ML) algorithms (decision trees, logistic regression, random forests, support vector machines (SVMs), extreme gradient boosting (XGBoost)). ML performance was compared with that of a deep-learning model based on the residual network (ResNet)-18 architecture using standard evaluation metrics such as the area under the curve (AUC).

**Results:**

In 2D TTE analysis, ResNet-18 achieved the highest AUC (0.889), followed by XGBoost (0.836) and decision tree (0.815). ResNet-18 significantly outperformed SVM (*P* = 0.013) and logistic regression (*P* = 0.028), but showed no significant differences versus XGBoost (*P* = 0.408), decision tree (*P* = 0.429), or random forest (*P* = 0.053). In 2D TEE analysis, SVM achieved the highest AUC (0.959), followed by XGBoost (0.924) and random forest (0.906), with no significant differences among these models (all *P* > 0.05). ResNet-18 (AUC = 0.900) significantly outperformed only the decision tree (*P* = 0.027).

**Conclusion:**

ResNet-18 showed the highest TTE AUC and outperformed SVM and logistic regression, but was comparable to other ML models. SVM achieved the highest TEE AUC, with no significant differences among top models. These findings provide a preliminary AI benchmark for right heart mass diagnosis, though external validation is needed.

**Supplementary Information:**

The online version contains supplementary material available at 10.1186/s12967-026-08322-8.

## Background

Right heart masses represent a histopathologically heterogeneous group, encompassing various etiologies including thrombi, and infective vegetations, as well as primary and metastatic neoplasms [1]. These masses lack pathognomonic clinical manifestations, rendering accurate preoperative characterization particularly challenging. Given the technical constraints and procedural risks associated with endomyocardial biopsy, cardiovascular imaging remains the cornerstone for differential diagnosis [2]. Although echocardiography serves as the primary imaging modality for lesion characterization, its diagnostic accuracy remains operator dependent.

Recent advances in computational power and data-processing techniques have propelled significant progress in artificial intelligence (AI) for medical image processing, demonstrating substantial clinical value. Using machine- and deep-learning algorithms, AI extracts radiomic features to capture lesion-specific biological and pathophysiological information. Through iterative model optimization, AI systems continuously improve diagnostic performance, showing particular promise in oncologic imaging applications [3, 4]. 

Ultrasound radiomics, in conjunction with machine-learning algorithms and deep convolutional neural networks, can quantify sonographic architectural and textural patterns, providing valuable clinical decision support for disease diagnosis, stratification, and prognostics [5–7]. Two-dimensional (2D) sonographic feature-based ultrasound radiomics has demonstrated significant clinical utility in diagnosing diseases of the superficial and abdominal organs, particularly excelling in the differential diagnosis of thyroid nodules and breast lesions [8, 9]. 

In recent years, the integration of AI with echocardiography has advanced rapidly in areas such as cardiac chamber quantification and disease classification. For instance, Hathaway et al. [10] developed a machine learning model using cardiac ultrasound images from 1915 subjects. External validation demonstrated that the model effectively predicted left ventricular remodeling, achieving areas under the curve (AUC) of 0.78 and 0.79 in point-of-care and high-end ultrasound system cohorts, respectively, with the predictions significantly associated with major adverse cardiovascular events. However, AI-based radiomics research in cardiac oncology remains limited. Bao et al. [11] analyzed echocardiographic data from 215 cardiac tumors in 121 patients and developed a classification nomogram by integrating clinical baseline characteristics with radiomics features. The model demonstrated performance comparable to that of senior physicians (AUC: 0.867 vs. 0.873, *P* = 0.928) and significantly outperformed junior physicians (AUC: 0.867 vs. 0.669, *P* = 0.029) in distinguishing benign from malignant cardiac tumors, providing preliminary evidence for the utility of ultrasound radiomics in cardiac tumor differentiation.

Therefore, to address inter-physician variability in echocardiography diagnosis and improve accuracy for challenging right heart masses, we developed AI-based binary classification models using radiomic features from 2D static transthoracic (TTE) and transesophageal (TEE) echocardiographic images using machine- and deep-learning approaches, identifying the best model using DeLong’s test.

## Methods

### Study design and patients

This single-center, retrospective study included 100 consecutive surgical patients with 103 right heart masses treated between January 2013 and March 2024. Preoperative 2D TTE and TEE echocardiographic images were acquired for each lesion. After excluding cases with incomplete data, the final analysis comprised 98 lesions with analyzable TTE images and 87 lesions with TEE images. The cohorts were stratified into a TTE group (*n* = 98) and a TEE group (*n* = 87), based on histopathology (Supplementary Table S1) as the gold standard. Clinical variables including age, sex, presenting symptoms, and history of malignancy were collected. A detailed analysis of clinical characteristics in this cohort is reported elsewhere (manuscript under review). This study was approved by the Ethics Committee of The First Affiliated Hospital, Zhejiang University School of Medicine (Approval No: [2025B] IIT-1225), in accordance with the Declaration of Helsinki.

### Protocol for echocardiographic image acquisition

Among the 100 enrolled patients, three had two discrete right heart masses, resulting in a total of 103 lesions analyzed. Each lesion was independently annotated. TTE was performed within one week before surgery for all patients. For TEE, 97 patients underwent intraoperative examination immediately after anesthesia induction under general anesthesia, while three patients underwent outpatient TEE within one week preoperatively with topical oral lidocaine gel anesthesia.

All TTE studies were performed using major ultrasound systems (including but not limited to the Philips IE33/EPIQ7 and GE VIVID E9 models) with appropriate transducers (S5-1/M5Sc/M5S) within one week preoperatively. TEE was conducted using Philips IE Elite/CX50 systems with X7-2t probes by board-certified cardiac sonographers.

### Image selection protocol

All echocardiographic images were retrospectively reviewed using the institutional ultrasound PACS workstation. Two blinded echocardiographers (each with over 10 years of experience in cardiac imaging) independently selected high-quality 2D static images of right cardiac masses according to the following predefined criteria: (1) clear image quality, (2) well-defined lesion edges, and (3) minimal artifacts. Discordant selections were resolved by consensus review. Images were exported in lossless BMP format to maintain diagnostic fidelity. This dual-reviewer system minimized individual operator bias in dataset construction.

Of the 103 lesions, TTE images were available for 98 lesions and TEE images for 87 lesions. Reasons for image exclusion included: (1) examinations performed at external hospitals without available images; (2) emergency bedside examinations where images were not stored; (3) images not retrievable from the PACS system despite extensive searches; and (4) poor image quality due to artifacts (e.g., electrocautery interference).

### Region of interest (ROI) delineation and image preprocessing

All echocardiographic images were exported from the PACS system in BMP format. During this export process, DICOM metadata—including pixel spacing information—were irretrievably lost. Therefore, spatial resampling to a uniform physical pixel size could not be performed.

**Fig. 1:**
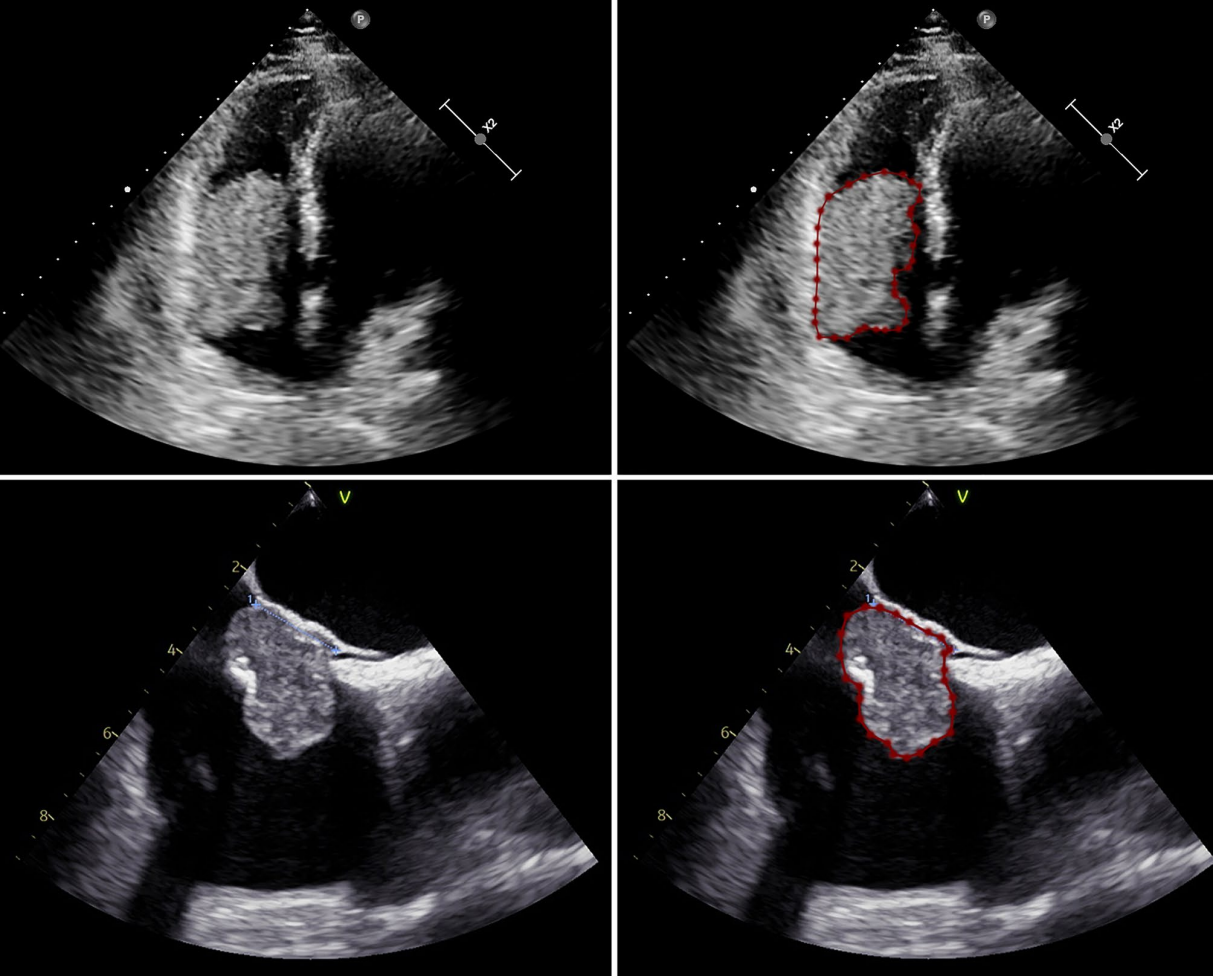
Region of interest labeling results of TTE and TEE 2D images

ROIs were manually delineated along the lesion boundaries by an expert echocardiographer (15 years of experience) using LabelMe (v5.1.1) [12], excluding non-imaging elements (e.g., calipers, patient IDs). The annotations fully encompassed the lesion margins (Fig. 1) and were exported as JSON coordinate sets.

Because the TTE and TEE images were acquired using multiple ultrasound systems with inconsistent probe settings, inevitable variations in resolution and contrast were present in the images. These factors could affect radiomic feature extraction in the ROIs. To address this, each image was independently normalized to a range of [0,1] using min-max normalization based on its own minimum and maximum pixel values. This approach preserves the relative contrast within each image while standardizing the intensity range across all images.

### Feature extraction and selection

For machine learning models, no image resizing was applied prior to feature extraction. Radiomic features were extracted directly from original images (preserving their original pixel dimensions and spatial resolution)using PyRadiomics (v3.0.1) [13, 14] following multiscale filtering. Features based on wavelet transforms, the Laplacian of Gaussian, and intensity transformations (logarithmic, square, square root) were extracted. Seven PyRadiomics feature classes were analyzed: “first-order” features, 2D morphological features, and texture analysis matrices (gray-level co-occurrence, run-length, size zone, dependence, neighboring gray-tone difference matrices).

Feature selection comprised three sequential steps: the elimination of low-variance features (variance < 0.0001), the removal of highly correlated features (Pearson’s *r* > 0.99), and final selection using LASSO regularization, which retains the most informative features via coefficient shrinkage.

### Dataset partition and cross-validation

In each image group, the dataset was partitioned into an independent test set (30%) and a training set (70%), with a consistent distribution of positive and negative samples across both sets. The test set was rigorously excluded from the model training process. Benign lesions accounted for 68.4% and 62.1% of the TTE and TEE 2D image groups, respectively, with non-benign lesions constituting the remainder.

To ensure rigorous model evaluation, five-fold cross-validation was implemented. Each fold preserved the original dataset’s class distribution, minimizing imbalance-related bias. The dataset was divided into five non-overlapping subsets. During each of the five iterations, a distinct subset was held out for validation, with the remaining subsets combined for training. Performance metrics from all iterations were aggregated to derive the final model assessment.

### Machine-learning model construction

This study employed five widely used machine-learning algorithms to develop binary classification models: decision trees, logistic regression, random forests, support vector machine (SVM), and extreme gradient boosting (XGBoost). Following generalization performance evaluation via cross-validation, the model with optimized hyperparameters was retrained on the entire training set. The final performance was rigorously assessed on the independent test set.

**To ensure fair comparison between models**,** all machine learning models underwent systematic** hyperparameter tuning using automated machine learning. Each model was trained for 200 iterations, and the optimal hyperparameters were selected based on AUC of validation from 5-fold cross-validation (n_repeats = 1). All experiments were conducted with a fixed random seed (random_seed = 0) to ensure reproducibility.

### Deep-learning model construction

Residual network (ResNet) [15], a deep-learning framework built on convolutional neural networks, was employed in this study. Based on the same manually delineated ROIs described above, the lesion regions were cropped from the original images, excluding interference from background areas. After cropping, the mean width and mean height of all ROI images were calculated. Based on these statistical findings, all ROIs were resized to a uniform size of 929 × 929 pixels (the calculated mean dimensions). This approach ensures that the standardized size reflects the statistical characteristics of the original data rather than being arbitrarily chosen. Subsequently, to meet the fixed input requirements of the ResNet, these 929 × 929 ROI images were further resized to 224 × 224 pixels. During this process, the original aspect ratio was not preserved, and no padding was applied. During model training, data augmentation techniques including random vertical and horizontal flipping (each with 50% probability) and translation were applied to improve model generalization.

Like the machine-learning models, the deep-learning model was subjected to stratified 5-fold cross-validation, with the test set reserved for evaluating the final trained model. The Adam optimizer was employed with cross-entropy loss as the objective function, along with an early stopping mechanism to mitigate overfitting. All experiments were conducted using PyTorch 1.13.1 and Python 3.10.12.

### Implementation details of the ResNet18 model

Among the evaluated architectures (ResNet18, ResNet34, and ResNet50) applied to the preprocessed TTE and TEE 2D images, ResNet18 demonstrated superior classification performance. The model training protocol incorporated an early stopping criterion that terminated training when the loss reduction remained below 0.001 for eight consecutive epochs. All experiments were conducted using an NVIDIA GeForce RTX 4090 GPU (24GB VRAM) with CUDA acceleration.

The ResNet-18 model was trained with the following hyperparameters: batch size = 8, maximum epochs = 50, learning rate = 0.001, using CUDA acceleration for binary classification (n_classes = 2) on a single GPU (num_gpu = 1), with random seed fixed at 7 (random_state = 7) to ensure reproducibility. Through validation, the optimal number of training epochs was determined to be 20 for the TTE cohort and 50 for the TEE cohort.

### Evaluation methods and metrics for model performance

This study comprehensively assessed model performance using seven widely-adopted quantitative metrics: AUC, accuracy (ACC), sensitivity (SEN), specificity (SPE), positive predictive value (PPV), negative predictive value (NPV), and the F1-score.

### Model prediction waterfall plot

This study employed waterfall plots to visualize model predictions across classification thresholds. For binary classification, the models output probability scores (0.000–1.000) representing positive-case likelihood. The optimal threshold was defined as the point nearest to (0.0, 1.0) on the average validation-set receiver operating characteristic (ROC) curve. This point maximizes the true positive rate while minimizing the false positive rate.

In a waterfall plot, the y-axis shows the predicted probabilities (0–1). It also shows the threshold as a horizontal line to indicate the optimal cutoff. Samples are arranged by ascending probability, and the true negative and true positive bars based on the ground truth are color coded.

### Statistical analysis

The continuous variables were analyzed using Student’s t-test or the Mann–Whitney U test, depending on the distribution characteristics. Categorical variables were evaluated using the Chi-square test. Statistical significance was defined as *P* < 0.05 (two-tailed).

To assess the predictive performance of AI models in distinguishing benign from non-benign right cardiac masses, the ROC curve was analyzed. Predictive accuracy was quantified using the AUC and reported with 95% confidence intervals (CI). Model AUC differences were assessed via DeLong’s test and reported with 95% CI. Inter-model comparisons were further assessed using the Z-statistic, reported as Z-values. All comparisons were deemed statistically significant at *P* < 0.05. All statistical analyses were performed using SPSS 26.0, R 4.3.2, and MedCalc 20.010.

## Results

### Patient group characteristics

The anatomical distribution of the 103 right heart masses is summarized in Supplementary Table S2, with the right atrium being the most common location (72.8%). Following the application of the inclusion and exclusion criteria, the final cohorts comprised 98 lesions in the TTE 2D image group and 87 lesions in the TEE 2D image group. For model development, the TTE training set contained 68 lesions, and the TEE training set contained 60 lesions. The numbers of positive cases (non-benign lesions) were 31 and 33 in the TTE and TEE groups, respectively.

### Feature extraction and selection results

Initial feature extraction yielded 846 features from both the TTE and TEE 2D images. Following feature selection, 167 features remained for the TTE images and 127 for the TEE images, including three morphological features for each modality. The texture features comprised the “first-order” features (36 for TTE; 33 for TEE), gray-level co-occurrence matrix (36 for TTE; 21 for TEE), gray-level run-length matrix (23 for TTE; 18 for TEE), gray-level size zone matrix (34 for TTE; 27 for TEE), neighboring gray-level difference matrix (17 for TTE; 11 for TEE), and gray-level dependence matrix (18 for TTE; 14 for TEE).

### Machine-learning model performance across study groups

Test set performance of AI models on TTE and TEE imags is presented in Tables 1 and 2, respectively. Complete training and validation metrics (mean ± standard deviation and 95% CIs for each evaluation metric by model type) are provided in Supplementary Table S3 and S4.

**Table 1:** Test set performance of various models on the TTE 2D image dataset

Models	AUC	ACC	SEN	SPE	PPV	NPV	F1 score
XGBoost	0.836	0.700	0.444	0.810	0.500	0.773	0.471
Decision trees	0.815	0.733	0.778	0.714	0.538	0.882	0.636
Random forests	0.714	0.733	0.222	0.952	0.667	0.741	0.333
SVM	0.635	0.733	0.222	0.952	0.667	0.741	0.333
Logistic regression	0.720	0.733	0.222	0.952	0.667	0.741	0.333
ResNet18	0.889	0.800	0.667	0.857	0.667	0.857	0.667

AUC: area under the receiver operating characteristic curve; ACC: accuracy; SEN: sensitivity; SPE: specificity; PPV: positive predictive value; NPV: negative predictive value

**Table 2:** Test set performance of various models on the TEE 2D image dataset

Models	AUC	ACC	SEN	SPE	PPV	NPV	F1 score
XGBoost	0.924	0.815	0.900	0.765	0.692	0.929	0.783
Decision trees	0.882	0.852	1.000	0.765	0.714	1.000	0.833
Random forests	0.906	0.815	0.800	0.824	0.727	0.875	0.762
SVM	0.959	0.852	0.800	0.882	0.800	0.882	0.800
Logistic regression	0.853	0.778	0.700	0.824	0.700	0.824	0.700
ResNet18	0.900	0.704	0.900	0.588	0.562	0.909	0.692

AUC: area under the receiver operating characteristic curve; ACC: accuracy; SEN: sensitivity; SPE: specificity; PPV: positive predictive value; NPV: negative predictive value

#### TTE 2D image analysis

The models demonstrated moderate discriminative ability (AUC range: 0.635–0.836) with consistent accuracy (ACC: 0.700–0.733). The decision tree model showed the highest sensitivity (77.8%) and negative predictive value (88.2%), while the random forest, SVM and logistic regression models achieved higher specificity (95.2% for all). The F1-scores were generally modest (maximum: 0.636 for the decision tree).

#### TEE 2D image analysis

The models achieved AUC values ranging from 0.853 to 0.959, with ACC ranging from 0.778 to 0.852. The decision tree and SVM models obtained the highest accuracy (both 0.852), with SVM demonstrating the highest specificity (88.2%) and positive predictive value (80.0%). The decision tree model achieved the highest sensitivity and F1-score (0.833). The negative predictive values ranged from 82.4% to 100%.

#### Visualization and comparative performance analysis

**Fig. 2:**
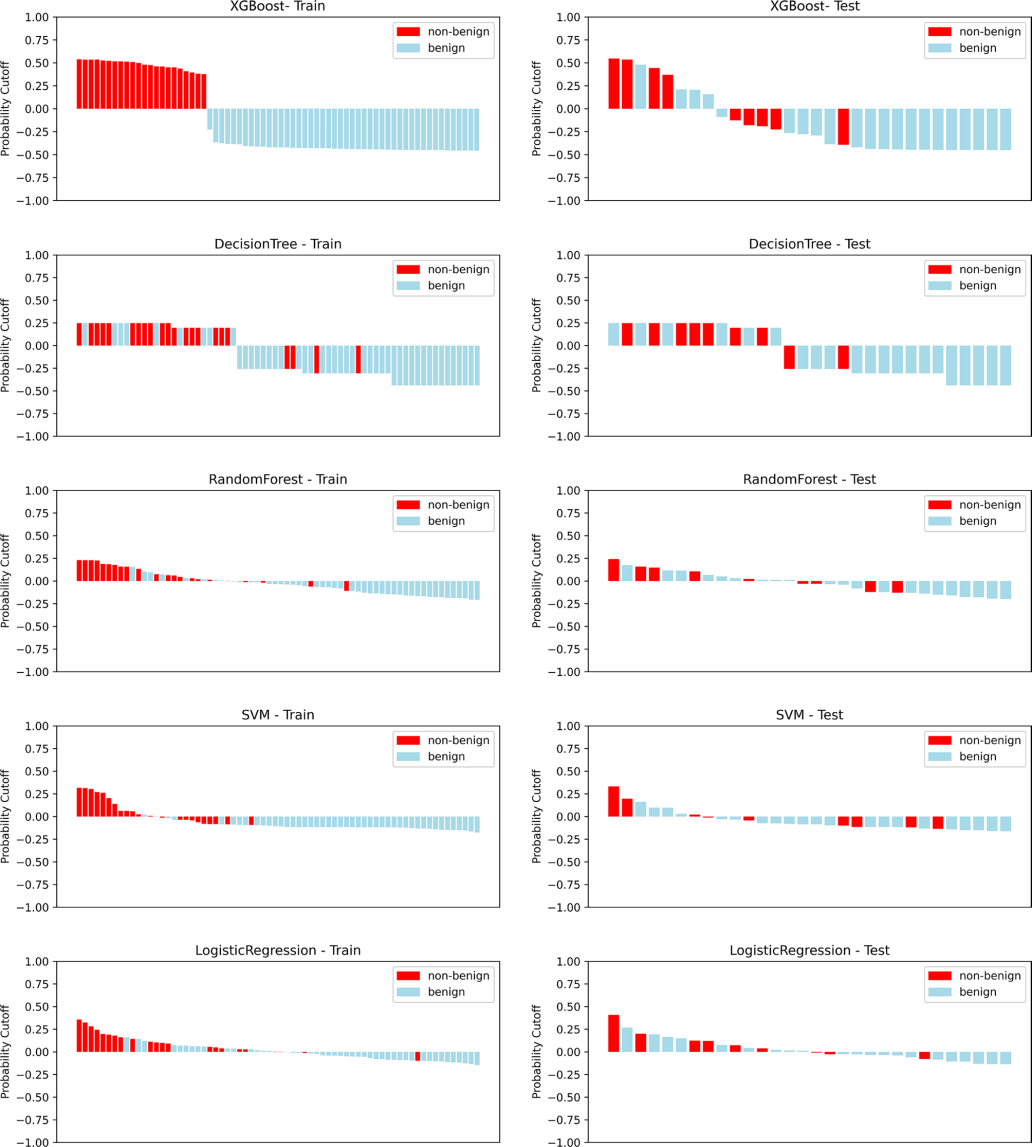
Training and test set waterfall plots for each machine learning model in the TTE 2D image group

**Fig. 3:**
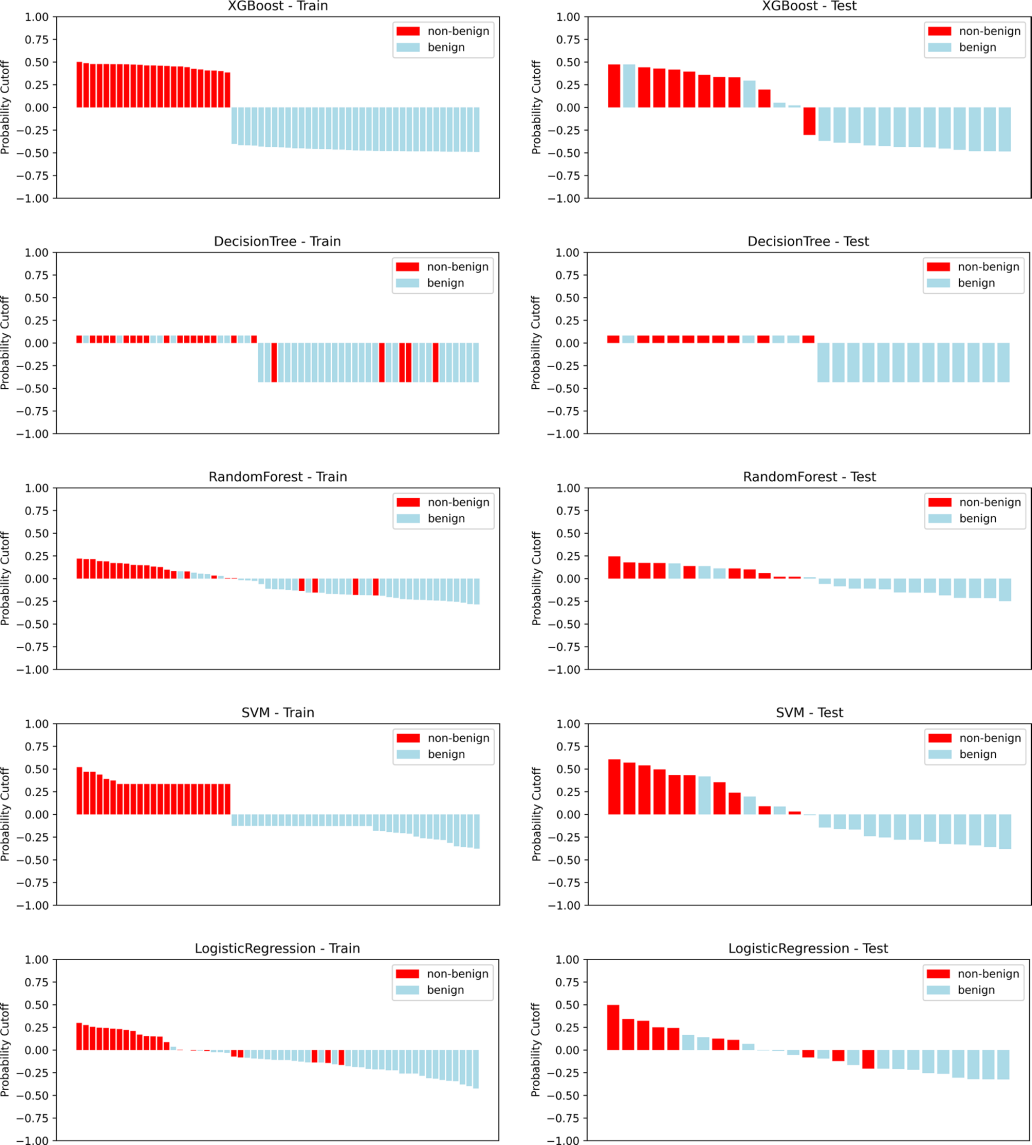
Training and test set waterfall plots for each machine learning model in the TEE 2D image group

The predictive performance of all models across the study groups was further visualized using waterfall plots (Figs. 2 and 3), which show the classification outcomes at different probability thresholds for both the training and test sets. The machine-learning models showed measurable ability in differentiating benign versus non-benign right heart masses.

### Comparative ROC analysis of machine-learning models

**Fig. 4:**
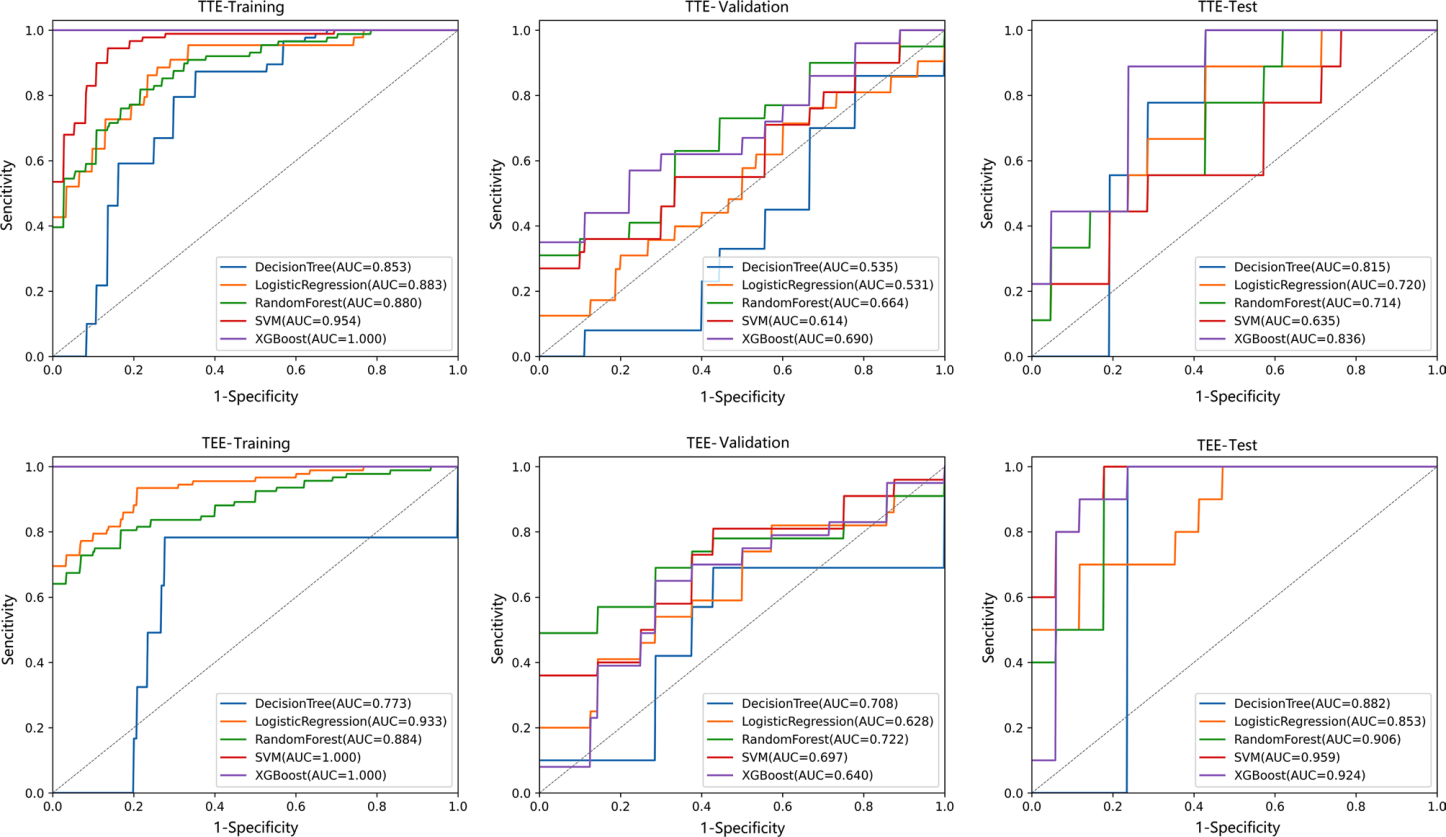
Comparison of ROC curves for training, validation, and test sets of various machine learning models on the TTE and TEE datasets

Figure 4 presents the ROC curves for all models across the training, validation, and test sets. The key findings were as follows: For TTE-based models, the XGBoost algorithm achieved the highest AUC (test set AUC = 0.836), with a statistically significantly higher AUC compared to SVM (AUC difference = 0.201, 95% CI [0.048 to 0.354]; Z = 2.582, *P* = 0.010). No other intermodel comparisons achieved statistical significance.

Among TEE-based models, all algorithms showed high discrimination (test set AUC range: 0.853–0.959), with SVM exhibiting the highest numerical performance (AUC = 0.959). However, DeLong’s comparison test indicated no statistically significant differences between models (all *P* > 0.05).

### Decision curve analysis (DCA) of the machine-learning models

DCA assesses clinical utility by quantifying net benefit across probability thresholds while balancing false-positive and false-negative trade-offs [16]. This analytic method compares model predictions against two default strategies (classifying all lesions as non-benign or benign) to establish the optimal intervention thresholds for clinical decision-making.

**Fig. 5:**
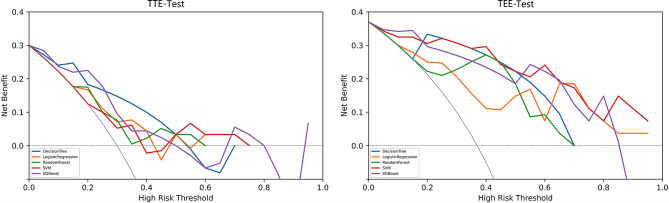
Decision curve analysis for various machine learning models in the TTE and TEE datasets

Our DCA of the test set data demonstrated that models developed from TEE images provided greater clinical net benefit than the TTE approaches (Fig. 5). The SVM algorithm achieved the highest net benefit for right cardiac mass classification on the TEE images.

### Performance of ResNet18 models on the 2D Static TTE and TEE images

For the ResNet18 model trained on 2D static TTE images, optimal performance was achieved at epoch 20 with a training set AUC of 0.932 (optimal threshold: 0.228). The test set evaluation obtained the following results: sensitivity 0.667, specificity 0.857, accuracy 0.800, F1-score 0.667, and AUC 0.889.

The ResNet18 model for the 2D static TEE images reached peak performance at epoch 50, attaining a training set AUC of 0.953 (optimal threshold: 0.775). Test set performance metrics included an AUC of 0.900, with corresponding sensitivity (0.900), specificity (0.588), accuracy (0.704), and F1-score (0.692).

**Fig. 6:**
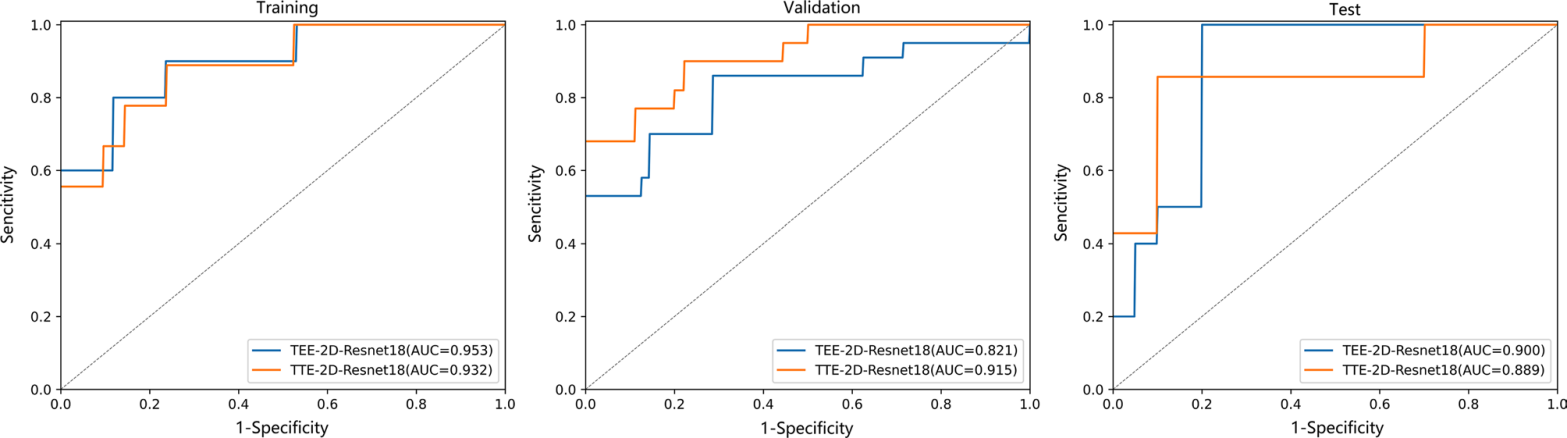
Comparison of ROC curves for training, validation, and test sets of deep learning models on the TTE and TEE datasets

**Fig. 7:**
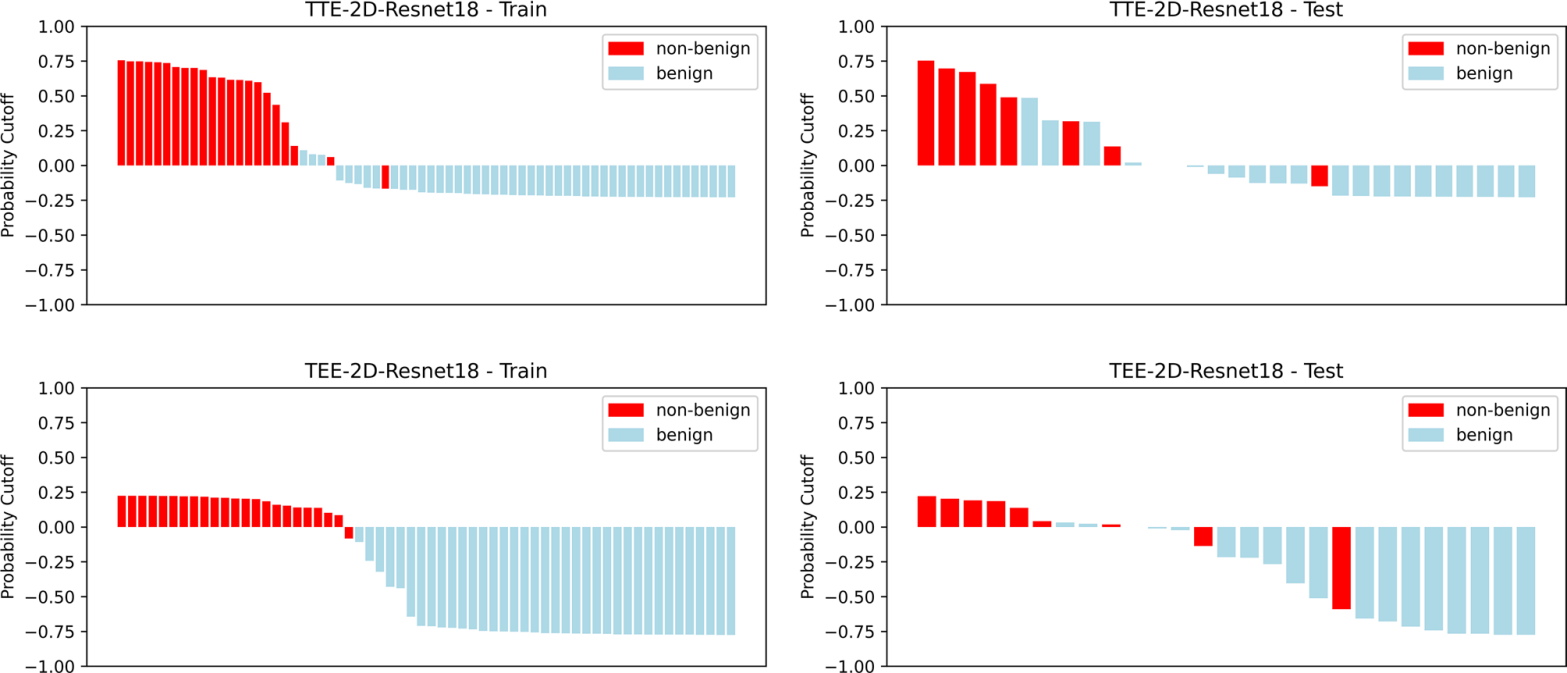
Waterfall plots of the ResNet18 model from the TTE and TEE 2D image datasets

**Fig. 8:**
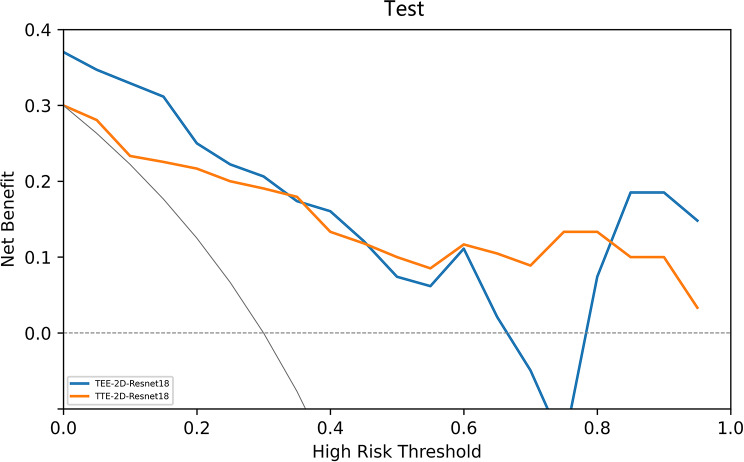
Decision curve analysis of the ResNet18 model on the TTE and TEE 2D image datasets

Figure 6 presents the ROC curves for both ResNet18 models. Additionally, waterfall plots illustrating the binary classification outcomes are provided in Fig. 7. Collectively, these results demonstrate that both TTE- and TEE-based ResNet18 models exhibit clinically relevant classification performance. The clinical net benefit of these models was further evidence using DCA (Fig. 8).

### Comparative analysis of the ResNet18 and machine-learning models on the 2D TTE and TEE images

On the TTE dataset, the ResNet18 model achieved an AUC of 0.889, which was numerically higher than the five machine learning algorithms. DeLong tests revealed that the AUC of ResNet18 was significantly higher than that of SVM (AUC difference = 0.254, 95% CI [− 0.453 to − 0.055]; Z = − 2.496, *P* = 0.013) and logistic regression (AUC difference = 0.169, 95% CI [− 0.321 to − 0.018]; Z = − 2.192, *P* = 0.028). However, no statistically significant differences in AUC were observed between ResNet18 and XGBoost (*P* = 0.408), decision tree (*P* = 0.429), or random forest (*P* = 0.053). These findings indicate that in TTE image classification, the AUC of ResNet18 was higher than that of some machine learning algorithms (SVM and logistic regression), but was not significantly different from those of XGBoost, decision tree, or random forest.

On the TEE dataset, the ResNet18 model achieved an AUC of 0.890. DeLong tests showed that the AUC of ResNet18 was significantly higher than that of the decision tree model (AUC difference = 0.118, 95% CI [− 0.222 to − 0.014]; Z = − 2.219, *P* = 0.027), while the difference compared with logistic regression was borderline significant (AUC difference = 0.147, 95% CI [− 0.294 to 0.000]; Z = − 1.960, *P* = 0.050). No significant differences in AUC were observed between ResNet18 and XGBoost (*P* = 0.178), SVM (*P* = 0.205), or random forest (*P* = 0.106). Additionally, DCA revealed that the TEE-based ResNet18 model showed negative net benefit at threshold probabilities of 0.65–0.78, with a minimum of -0.15.

## Discussion

Traditional echocardiographic diagnosis relies heavily on physicians’ subjective visual assessments of morphological characteristics such as lesion shape, echogenicity, and margins [17]. In contrast, radiomics employs data-driven algorithms to perform the high-throughput extraction of subvisual features from images, including morphometric parameters, textural patterns, and signal intensity distribution. This quantitative approach enables features to be comprehensively characterized and the computational analysis of deep-layer image information that is imperceptible to human observers. By systematically decoding these high-dimensional imaging signatures, radiomics provides an objective decision-support tool that may enhance clinical decision-making processes [18, 19]. The results of this study demonstrate the feasibility of constructing AI models based on radiomic features extracted from 2D echocardiographic images for the classification of right cardiac masses. This approach has the potential to enhance diagnostic objectivity and reduce reliance on echocardiographer-dependent subjective interpretation.

Among the machine-learning models developed using TTE images, ensemble methods such as XGBoost demonstrated favorable performance. Notably, several models exhibited high specificity in identifying benign lesions, which may facilitate subsequent diagnostic decision-making. Comparative analysis revealed that the models constructed from TEE images consistently outperformed their TTE-based counterparts, suggesting that radiomic features extracted from TEE images may possess greater discriminative power for the machine-learning frameworks employed in this study.

Several factors may explain this observation. First, TEE’s proximity to cardiac structures avoids chest wall and lung interference, providing inherently higher resolution—particularly advantageous for visualizing small masses. Second, most TEE images were acquired intraoperatively under general anesthesia, ensuring stable image quality without motion artifacts or gag reflexes. Third, the higher exclusion rate for TEE images may have introduced selection bias, with the remaining images representing a higher-quality subset that could contribute to the observed performance advantage.

Although DeLong’s test did not reveal statistically significant differences among the TEE-based models, the SVM algorithm achieved the highest numerical test AUC (0.959). However, cross-validation results revealed a substantial discrepancy between training (AUC = 1.000) and validation (AUC = 0.697 ± 0.214) performance, indicating potential overfitting. This observation underscores the importance of cautious interpretation of test set performance in small-sample settings and highlights the need for more robust evaluation strategies, such as repeated cross-validation or external validation, in future studies.

With this caveat in mind, the numerical performance of SVM on the TEE dateset is consistent with previous ultrasound radiomics studies that have reported favorable results with this algorithm. For instance, Vadhiraj et al. [20] achieved 96% accuracy in differentiating benign and malignant thyroid nodules using SVM with radiomic features extracted from 2D ultrasound images.

In contrast to the machine learning approaches discussed above, deep learning offers an alternative paradigm for feature learning. In the field of medical image processing, ResNet18 has been demonstrated to achieve reliable performance across multiple tasks [21, 22]. In this study, ResNet18 was selected as the deep learning architecture due to its good generalization capability and ability to learn from limited data-a critical advantage for small-sample tasks. As a lightweight residual network with a relatively low parameter count, ResNet18 may offer enhanced adaptability in small-sample scenarios.

In image recognition tasks, deep-learning models extract multi-level features from images through layered convolution and pooling operations, achieving high-precision recognition [23, 24]. These capabilities have demonstrated significant potential in ultrasound image analysis across various clinical applications. For instance, a multicenter study of 558 patients with pancreatic lesions developed a ResNet50-based model using contrast-enhanced ultrasound, achieving high diagnostic performance with AUCs of 0.986 in training, 0.978 in internal validation, and 0.967 and 0.953 in two external tests [25]. Similarly, deep-learning models based on ultrasound imaging have shown strong performance in the differential diagnosis of breast nodules, hepatic lesions, and prostatic abnormalities [26–28]. These findings highlight the promising clinical utility of deep-learning techniques in tumor characterization.

Consistent with the aforementioned studies, our results demonstrate that ResNet18 effectively extracted deep features from TTE 2D images, achieving an AUC of 0.889—the highest among all models on the TTE dataset. Notably, despite being more complex than machine learning algorithms, ResNet-18 exhibited better generalization capability on the TTE dataset. This may be attributed to several inherent factors in the deep learning training protocol: first, the early stopping mechanism effectively prevented overfitting; second, data augmentation expanded the effective sample size and improved model robustness; third, the end-to-end feature learning capability of deep learning models may capture more generalizable representations than handcrafted radiomic features, which are more sensitive to variations in acquisition conditions. As a lightweight residual network with a relatively low parameter count, ResNet18 may further enhance its adaptability in small-sample scenarios, a characteristic particularly valuable for research on rare conditions such as right heart masses.

The AI models developed in this study have several potential clinical applications. First, they could serve as a triage tool to identify patients with suspected malignant right heart masses, prioritizing them for further advanced imaging such as cardiac magnetic resonance or positron emission tomography. Second, in resource-limited settings or for less experienced physicians, these models could act as a diagnostic aid, providing real-time risk assessment during echocardiographic examinations. It is important to emphasize that these AI tools are intended to assist, not replace, clinical decision-making, and their integration into routine practice would require prospective validation and careful consideration of workflow integration.

### Limitations

This study has several important limitations that should be acknowledged. First, its single-center, retrospective design with a relatively small sample size is the most significant limitation. Although TEE-based models achieved high AUC values (> 0.90), these results require external validation in multi-center prospective cohorts before clinical application. Second, the limited sample size contributed to overfitting in some machine learning models, as evidenced by near-perfect training AUC but substantially lower validation/test AUC. This highlights the need for validation in larger cohorts. Future studies should incorporate repeated cross-validation for more robust performance estimates. Third, all images were exported in BMP format, resulting in loss of DICOM metadata including pixel spacing information. Consequently, spatial resampling to uniform physical pixel size could not be performed, which may affect the reliability of shape- and size-related features. Future studies should store DICOM images to enable spatial resolution normalization, and could further optimize model performance by: (1) incorporating absolute size information; (2) including anatomical location as an auxiliary feature; (3) exploring attention mechanisms or multi-scale inputs that preserve both lesion features and surrounding anatomical context. Fourth, the discrepancy in image availability between TTE (98 lesions) and TEE (87 lesions) may have introduced selection bias, potentially overestimating TEE model performance. Fifth, decision curve analysis revealed that the TEE-based ResNet-18 model showed negative net benefit at threshold probabilities of 0.65–0.78, indicating caution is warranted when applying this model in conservative clinical decision-making contexts. Sixth, although we applied per-image normalization and feature selection (reducing features from 846 to 167 for TTE and 127 for TEE), we did not employ harmonization methods such as ComBat to correct for potential inter-device differences. Such methods are more suitable for future multi-center studies. Seventh, clinical variables were not incorporated into the models, as this study focused on pure ultrasound image features. A separate analysis of clinical characteristics is currently under review. Future studies should integrate imaging features with clinical data to develop multi-modal models that better align with real-world diagnostic workflows. With larger multi-center cohorts, it will also be possible to perform subgroup analyses to understand model performance across different pathological entities and clinical presentations.

## Conclusion

In this pilot study, ResNet-18 achieved the highest numerical AUC in TTE classification and significantly outperformed SVM and logistic regression, but showed comparable performance to XGBoost, decision tree, and random forest. In TEE analysis, SVM achieved the highest numerical AUC, with no statistically significant differences among the top-performing machine learning models. These findings provide a preliminary AI benchmark for right heart mass diagnosis using echocardiographic images. However, given the single-center, retrospective design and relatively small sample size, all results should be considered preliminary. External validation in multi-center, prospective cohorts is essential before clinical application.

## Electronic Supplementary Material

Below is the link to the electronic supplementary material.

Supplementary Material 1

## Data Availability

All data generated or analysed during this study are included in this published article and its supplementary information files.

## Abbreviations

AI: Artificial intelligence
2D: Two dimension
TTE: Transthoracic echocardiography
TEE: Transesophageal echocardiography
ROI: Region of interest
SVM: Support vector machine
XGBoost: Extreme gradient boosting
ResNet: Residual network
AUC: Area under curve
ACC: Accuracy
SEN: Sensitivity
SPE: Specificity
PPV: Positive predictive value
NPV: Negative predictive value
ROC: Receiver operating characteristic
STD: Standard Deviation
CI: Confidence intervals
DCA: Decision curve analysis
