# Supplementary material for: AI-assisted radiomics for classification of benign and non-benign right heart masses in 2D-echocardiography
Source: J Transl Med. 2026 May 23;24:964. doi: 10.1186/s12967-026-08322-8 (PMC13412137; doi:10.1186/s12967-026-08322-8)
Supplement: Supplementary file 1 — Supplementary Material 1 [file 12967_2026_8322_MOESM1_ESM.doc]

| **S1. The pathological types of all masses** | | | |
| --- | --- | --- | --- |
| **Pathological types** | Lesions | **TTE** | **TEE** |
| **Non-benign masses** | **34（33.01%）** | **31** | **33** |
| **Angiosarcoma** | **9（26.47%）** | **9** | **8** |
| **Non-Hodgkin lymphoma** | **7（20.59%）** | **7** | **7** |
| **Metastatic tumors** | **12（35.29%）** | **10** | **12** |
| **Leiomyosarcoma** | **1（2.94%）** | **0** | **1** |
| **Malignant Mesothelioma** | **1（2.94%）** | **1** | **1** |
| **Low-grade malignant vascular tumor** | **1（2.94%）** | **1** | **1** |
| **Lipogenic Tumors** | **1（2.94%）** | **1** | **1** |
| **Inflammatory Myofibroblastic Tumor** | **1（2.94%）** | **1** | **1** |
| **Histiocytosis** | **1（2.94%）** | **1** | **1** |
| **Benign masses** | **69（66.99%）** | **67** | **54** |
| **Myxoma** | **36（52.17%）** | **35** | **24** |
| **Thrombus** | **8（11.59%）** | **8** | **7** |
| **Lipoma** | **6（8.70%）** | **6** | **5** |
| **Hemangioma** | **6（8.70%）** | **6** | **5** |
| **Cyst** | **3（4.35%）** | **3** | **3** |
| **Calcifying Amorphous Tumor** | **1（1.45%）** | **1** | **1** |
| **Leiomyoma** | **1（1.45%）** | **1** | **1** |
| **Extensively Necrotic and Calcified Tumor Tissue** | **1（1.45%）** | **1** | **1** |
| **Intravenous Leiomyomatosis** | **1（1.45%）** | **1** | **1** |
| **Papillary Fibroelastoma** | **1（1.45%）** | **1** | **1** |
| **Pericardial Calcification** | **1（1.45%）** | **1** | **1** |
| **Fibrous Tissue Hyperplasia, Collagenization with Calcification** | **2（2.90%）** | **1** | **2** |
| **Fibrinoid Necrosis with Calcification** | **1（1.45%）** | **1** | **1** |
| **Cystic Wall-like Tissue, Fibrous Hyperplasia, Collagenization with Thrombosis** | **1（1.45%）** | **1** | **1** |
| **Total** | **103** | **98** | **87** |
| **Representation of the Composition of Tumors by Frequency (Percentage)** | | | |

|  |
| --- |

|  |
| --- |

| S2. Anatomical distribution of right heart masses | | | | |
| --- | --- | --- | --- | --- |
|  |  | Right heart masses | |  |
|  |  | Benign | Non-benign |  |
|  | Total，N = 103^1^ | N = 69 (67%)^1^ | N = 34 (33%)^1^ | P-value |
| Location |  |  |  | 0.009 |
| Right atrium | 75 (72.82%) | 56 (81.16%) | 19 (55.88%) |  |
| Right atrium and ventricle | 2 (1.94%) | 0 (0.00%) | 2 (5.88%) |  |
| Right ventricle | 13 (12.62%) | 7 (10.14%) | 6 (17.65%) |  |
| Right ventricle and pulmonary artery | 1 (0.97%) | 1 (1.45%) | 0 (0.00%) |  |
| Tricuspid valve | 4 (3.88%) | 3 (4.35%) | 1 (2.94%) |  |
| Pericardium (adjacent to right heart) | 2 (1.94%) | 1 (1.45%) | 1 (2.94%) |  |
| Extension from vena cava to right heart | 6 (5.83%) | 1 (1.45%) | 5 (14.71%) |  |
| ^1^ Values are presented as n (%) |  |  |  |  |

| **S3. Performance of Various Models on the TTE 2D Image Dataset** | | | | | | | | |
| --- | --- | --- | --- | --- | --- | --- | --- | --- |
| **TTE Models** | **Datasets** | **AUC ± STD**  **(95% CI)** | **ACC ± STD**  **(95% CI)** | **SEN ± STD**  **(95% CI)** | **SPE ± STD**  **(95% CI)** | **PPV ± STD**  **(95% CI)** | **NPV ± STD**  **(95% CI)** | **F1 ± STD**  **(95% CI)** |
| **XGBoost** | **Training** | 1.000 ± 0.000 (1.000 ， 1.000) | 1.000 ± 0.000 (1.000 ， 1.000) | 1.000 ± 0.000 (1.000 ， 1.000) | 1.000 ± 0.000 (1.000 ， 1.000) | 1.000 ± 0.000 (1.000 ， 1.000) | 1.000 ± 0.000 (1.000 ， 1.000) | 1.000 ± 0.000 (1.000 ， 1.000) |
|  | **Validation** | 0.690 ± 0.149 (0.483 ， 0.897) | 0.722 ± 0.113 (0.566 ， 0.878) | 0.440 ± 0.203 (0.158 ， 0.722) | 0.851 ± 0.103 (0.708 ， 0.994) | 0.610 ± 0.262 (0.247 ， 0.973) | 0.767 ± 0.075 (0.663 ， 0.871) | 0.499 ± 0.205 (0.214 ， 0.784) |
|  | **Test** | 0.836 | 0.700 | 0.444 | 0.810 | 0.500 | 0.773 | 0.471 |
| **Decision trees** | **Training** | 0.853 ± 0.021 (0.824 ， 0.883) | 0.787 ± 0.014 (0.767 ， 0.806) | 0.669 ± 0.117 (0.507 ， 0.831) | 0.842 ± 0.049 (0.774 ， 0.910) | 0.675 ± 0.030 (0.633 ， 0.717) | 0.847 ± 0.044 (0.786 ， 0.909) | 0.666 ± 0.047 (0.601 ， 0.730) |
|  | **Validation** | 0.535 ± 0.115 (0.375 ， 0.695) | 0.527 ± 0.107 (0.379 ， 0.676) | 0.330 ± 0.264 (-0.036， 0.696) | 0.638 ± 0.225 (0.325 ， 0.950) | 0.327 ± 0.183 (0.073 ， 0.581) | 0.671 ± 0.066 (0.580 ， 0.762) | 0.280 ± 0.152 (0.070 ， 0.490) |
|  | **Test** | 0.815 | 0.733 | 0.778 | 0.714 | 0.538 | 0.882 | 0.636 |

**S3. Performance of Various Models on the TTE 2D Image Dataset (continued)**

| **TTE Models** | **Datasets** | **AUC ± STD**  **(95% CI)** | **ACC ± STD**  **(95% CI)** | **SEN ± STD**  **(95% CI)** | **SPE ± STD**  **(95% CI)** | **PPV ± STD**  **(95% CI)** | **NPV ± STD**  **(95% CI)** | **F1 ± STD**  **(95% CI)** |
| --- | --- | --- | --- | --- | --- | --- | --- | --- |
| **Random forests** | **Training** | 0.880 ± 0.014 (0.861 ， 0.899) | 0.739 ± 0.014 (0.719 ， 0.759) | 0.192 ± 0.054 (0.117 ， 0.267) | 1.000 ± 0.000 (1.000 ， 1.000) | 1.000 ± 0.000 (1.000 ， 1.000) | 0.722 ± 0.011 (0.707 ， 0.736) | 0.319 ± 0.076 (0.214 ， 0.424) |
|  | **Validation** | 0.664 ± 0.069 (0.569 ， 0.760) | 0.736 ± 0.072 (0.637 ， 0.836) | 0.190 ± 0.185 (-0.067， 0.447) | 1.000 ± 0.000 (1.000 ， 1.000) | 0.600 ± 0.490 (-0.080， 1.280) | 0.723 ± 0.062 (0.636 ， 0.810) | 0.280 ± 0.254 (-0.073， 0.633) |
|  | **Test** | 0.714 | 0.733 | 0.222 | 0.952 | 0.667 | 0.741 | 0.333 |
| **SVM** | **Training** | 0.954 ± 0.019 (0.928 ， 0.980) | 0.805 ± 0.018 (0.780 ， 0.831) | 0.397 ± 0.064 (0.307 ， 0.486) | 1.000 ± 0.000 (1.000 ， 1.000) | 1.000 ± 0.000 (1.000 ， 1.000) | 0.777 ± 0.015 (0.755 ， 0.798) | 0.565 ± 0.065 (0.475 ， 0.655) |
|  | **Validation** | 0.614 ± 0.060 (0.532 ， 0.697) | 0.722 ± 0.067 (0.628 ， 0.815) | 0.330 ± 0.140 (0.136 ， 0.524) | 0.918 ± 0.117 (0.755 ， 1.080) | 0.780 ± 0.271 (0.403 ， 1.157) | 0.742 ± 0.049 (0.673 ， 0.810) | 0.422 ± 0.130 (0.242 ， 0.602) |
|  | **Test** | 0.635 | 0.733 | 0.222 | 0.952 | 0.667 | 0.741 | 0.333 |

**S3. Performance of Various Models on the TTE 2D Image Dataset (continued)**

| **TTE Models** | | **Datasets** | | **AUC ± STD**  **(95% CI)** | | **ACC ± STD**  **(95% CI)** | | **SEN ± STD**  **(95% CI)** | | **SPE ± STD**  **(95% CI)** | | **PPV ± STD**  **(95% CI)** | | **NPV ± STD**  **(95% CI)** | | **F1 ± STD**  **(95% CI)** |
| --- | --- | --- | --- | --- | --- | --- | --- | --- | --- | --- | --- | --- | --- | --- | --- | --- |
| **Logistic regression** | | **Training** | | 0.883 ± 0.037 (0.772 ， 0.995) | | 0.735 ± 0.018 (0.679 ， 0.791) | | 0.179 ± 0.081 (-0.067， 0.426) | | 1.000 ± 0.000 (1.000 ， 1.000) | | 1.000 ± 0.000 (1.000 ， 1.000) | | 0.719 ± 0.011 (0.685 ， 0.753) | | 0.296 ± 0.120 (-0.070， 0.662) |
|  |  | **Validation** | | 0.531 ± 0.175 (-0.001， 1.063) | | 0.648 ± 0.030 (0.556 ， 0.739) | | 0.048 ± 0.067 (-0.157， 0.253) | | 0.938 ± 0.088 (0.669 ， 1.206) | | 0.083 ± 0.118 (-0.275， 0.442) | | 0.673 ± 0.015 (0.628 ， 0.717) | | 0.061 ± 0.086 (-0.200， 0.321) |
|  |  | **Test** | | 0.720 | | 0.733 | | 0.222 | | 0.952 | | 0.667 | | 0.741 | | 0.333 |
| **ResNet18** | | **Training** | | 0.973 ± 0.024 (0.939 ， 1.007) | | 0.934 ± 0.053 (0.861 ， 1.007) | | 0.896 ± 0.101 (0.755 ， 1.037) | | 0.951 ± 0.046 (0.887 ， 1.016) | | 0.901 ± 0.083 (0.785 ， 1.016) | | 0.952 ± 0.043 (0.893 ， 1.012) | | 0.896 ± 0.085 (0.779 ， 1.013) |
|  | | **Validation** | | 0.915 ± 0.064 (0.827 ， 1.003) | | 0.824 ± 0.112 (0.669 ， 0.979) | | 0.780 ± 0.204 (0.497 ， 1.063) | | 0.849 ± 0.165 (0.620 ， 1.078) | | 0.760 ± 0.224 (0.448 ， 1.072) | | 0.901 ± 0.086 (0.782 ， 1.021) | | 0.743 ± 0.164 (0.516 ， 0.971) |
|  | | **Test** | | 0.889 | | 0.800 | | 0.667 | | 0.857 | | 0.667 | | 0.857 | | 0.667 |
| TTE Transthoracic echocardiography, SVM Support vector machine, XGBoost Extreme gradient boosting, ResNet Residual network, AUC Area under curve, ACC Accuracy, SEN Sensitivity, SPE Specificity, PPV Positive predictive value, NPV Negative predictive value, STD Standard Deviation,CI Confidence intervals | | | | | | | | | | | | | | | | |
| **S4. Performance of Various Models on the TEE 2D Image Dataset** | | | | | | | | | | | | | | | | |
| **TEE Models** | **Datasets** | | **AUC ± STD**  **(95% CI)** | | **ACC ± STD**  **(95% CI)** | | **SEN ± STD**  **(95% CI)** | | **SPE ± STD**  **(95% CI)** | | **PPV ± STD**  **(95% CI)** | | **NPV ± STD**  **(95% CI)** | | **F1 ± STD**  **(95% CI)** | |
| **XGBoost** | **Training** | | 1.000 ± 0.000 (1.000 ， 1.000) | | 1.000 ± 0.000 (1.000 ， 1.000) | | 1.000 ± 0.000 (1.000 ， 1.000) | | 1.000 ± 0.000 (1.000 ， 1.000) | | 1.000 ± 0.000 (1.000 ， 1.000) | | 1.000 ± 0.000 (1.000 ， 1.000) | | 1.000 ± 0.000 (1.000 ， 1.000) | |
|  | **Validation** | | 0.640 ± 0.174 (0.398 ， 0.881) | | 0.600 ± 0.097 (0.465 ， 0.735) | | 0.410 ± 0.304 (-0.012， 0.832) | | 0.732 ± 0.148 (0.527 ， 0.938) | | 0.528 ± 0.252 (0.178 ， 0.877) | | 0.686 ± 0.162 (0.461 ， 0.911) | | 0.399 ± 0.172 (0.161 ， 0.637) | |
|  | **Test** | | 0.924 | | 0.815 | | 0.900 | | 0.765 | | 0.692 | | 0.929 | | 0.783 | |
| **Decision trees** | **Training** | | 0.773 ± 0.037 (0.722 ， 0.825) | | 0.771 ± 0.035 (0.722 ， 0.819) | | 0.783 ± 0.047 (0.718 ， 0.848) | | 0.763 ± 0.031 (0.721 ， 0.806) | | 0.673 ± 0.039 (0.619 ， 0.728) | | 0.850 ± 0.034 (0.803 ， 0.897) | | 0.724 ± 0.041 (0.667 ， 0.781) | |
|  | **Validation** | | 0.708 ± 0.092 (0.580 ， 0.835) | | 0.717 ± 0.100 (0.578 ， 0.855) | | 0.690 ± 0.174 (0.448 ， 0.932) | | 0.725 ± 0.148 (0.520 ， 0.930) | | 0.663 ± 0.186 (0.404 ， 0.921) | | 0.803 ± 0.115 (0.643 ， 0.963) | | 0.649 ± 0.100 (0.511 ， 0.788) | |
|  | **Test** | | 0.882 | | 0.852 | | 1.000 | | 0.765 | | 0.714 | | 1.000 | | 0.833 | |

**S4. Performance of Various Models on the TEE 2D Image Dataset (continued)**

| **TEE Models** | **Datasets** | **AUC ± STD**  **(95% CI)** | **ACC ± STD**  **(95% CI)** | **SEN ± STD**  **(95% CI)** | **SPE ± STD**  **(95% CI)** | **PPV ± STD**  **(95% CI)** | **NPV ± STD**  **(95% CI)** | **F1 ± STD**  **(95% CI)** |
| --- | --- | --- | --- | --- | --- | --- | --- | --- |
| **Random forests** | **Training** | 0.884 ± 0.017 (0.860 ， 0.908) | 0.846 ± 0.039 (0.792 ， 0.899) | 0.663 ± 0.125 (0.490 ， 0.836) | 0.959 ± 0.034 (0.912 ， 1.006) | 0.919 ± 0.054 (0.844 ， 0.994) | 0.825 ± 0.051 (0.754 ， 0.896) | 0.761 ± 0.080 (0.650 ， 0.872) |
|  | **Validation** | 0.722 ± 0.120 (0.556 ， 0.888) | 0.683 ± 0.033 (0.637 ， 0.730) | 0.400 ± 0.192 (0.133 ， 0.667) | 0.868 ± 0.137 (0.677 ， 1.058) | 0.767 ± 0.200 (0.489 ， 1.044) | 0.706 ± 0.070 (0.609 ， 0.803) | 0.467 ± 0.092 (0.339 ， 0.594) |
|  | **Test** | 0.906 | 0.815 | 0.800 | 0.824 | 0.727 | 0.875 | 0.762 |
| **SVM** | **Training** | 1.000 ± 0.000 (1.000 ， 1.000) | 0.938 ± 0.125 (0.764 ， 1.111) | 0.833 ± 0.333 (0.371 ， 1.296) | 1.000 ± 0.000 (1.000 ， 1.000) | 1.000 ± 0.000 (1.000 ， 1.000) | 0.933 ± 0.133 (0.748 ， 1.118) | 0.857 ± 0.286 (0.461 ， 1.254) |
|  | **Validation** | 0.697 ± 0.214 (0.400 ， 0.994) | 0.617 ± 0.085 (0.499 ， 0.735) | 0.260 ± 0.174 (0.018 ， 0.502) | 0.843 ± 0.151 (0.633 ， 1.053) | 0.547 ± 0.394 (-0.001， 1.094) | 0.649 ± 0.056 (0.570 ， 0.727) | 0.320 ± 0.193 (0.052 ， 0.588) |
|  | **Test** | 0.959 | 0.852 | 0.800 | 0.882 | 0.800 | 0.882 | 0.800 |

**S4. Performance of Various Models on the TEE 2D Image Dataset (continued)**

| **TEE Models** | **Datasets** | **AUC ± STD**  **(95% CI)** | **ACC ± STD**  **(95% CI)** | **SEN ± STD**  **(95% CI)** | **SPE ± STD**  **(95% CI)** | **PPV ± STD**  **(95% CI)** | **NPV ± STD**  **(95% CI)** | **F1 ± STD**  **(95% CI)** |
| --- | --- | --- | --- | --- | --- | --- | --- | --- |
| **Logistic regression** | **Training** | 0.933 ± 0.021 (0.904 ， 0.963) | 0.871 ± 0.020 (0.842 ， 0.899) | 0.695 ± 0.046 (0.632 ， 0.759) | 0.980 ± 0.027 (0.943 ， 1.017) | 0.959 ± 0.054 (0.884 ， 1.034) | 0.839 ± 0.018 (0.813 ， 0.864) | 0.804 ± 0.034 (0.757 ， 0.851) |
|  | **Validation** | 0.628 ± 0.152 (0.417 ， 0.839) | 0.633 ± 0.067 (0.541 ， 0.726) | 0.450 ± 0.179 (0.202 ， 0.698) | 0.768 ± 0.180 (0.518 ， 1.018) | 0.633 ± 0.221 (0.326 ， 0.940) | 0.694 ± 0.071 (0.596 ， 0.792) | 0.467 ± 0.092 (0.339 ， 0.594) |
|  | **Test** | 0.853 | 0.778 | 0.700 | 0.824 | 0.700 | 0.824 | 0.700 |
| **ResNet18** | **Training** | 0.882 ± 0.136 (0.693 ， 1.071) | 0.858 ± 0.131 (0.677 ， 1.040) | 0.761 ± 0.215 (0.463 ， 1.059) | 0.920 ± 0.100 (0.781 ， 1.059) | 0.856 ± 0.188 (0.595 ， 1.117) | 0.867 ± 0.116 (0.706 ， 1.029) | 0.797 ± 0.187 (0.537 ， 1.057) |
|  | **Validation** | 0.821 ± 0.085 (0.702 ， 0.940) | 0.833 ± 0.091 (0.707 ， 0.960) | 0.700 ± 0.266 (0.330 ， 1.070) | 0.918 ± 0.067 (0.824 ， 1.011) | 0.877 ± 0.104 (0.732 ， 1.021) | 0.851 ± 0.119 (0.687 ， 1.016) | 0.730 ± 0.205 (0.445 ， 1.015) |
|  | **Test** | 0.900 | 0.704 | 0.900 | 0.588 | 0.562 | 0.909 | 0.692 |
| TEE Transesophageal echocardiography, SVM Support vector machine, XGBoost Extreme gradient boosting, ResNet Residual network, AUC Area under curve, ACC Accuracy, SEN Sensitivity, SPE Specificity, PPV Positive predictive value, NPV Negative predictive value, STD Standard Deviation,CI Confidence intervals | | | | | | | | |
